# 643. Animal Bites Case Surveillance and Rabies Post Exposure Prophylaxis in the Republic of Korea Military, From 2012 to 2019

**DOI:** 10.1093/ofid/ofad500.707

**Published:** 2023-11-27

**Authors:** Hong Sang Oh, Se-yong Kim, Gyeong-Yong Sung

**Affiliations:** Hallym University Sacred Heart Hospital, Anyang-si, Kyonggi-do, Republic of Korea; Armed Forces Medical Command, Seongnam-si, Kyonggi-do, Republic of Korea; Ground Operations Command, Yong In-si, Kyonggi-do, Republic of Korea

## Abstract

**Background:**

Rabies is a fatal zoonosis on a global scale. In South Korea, no human rabies and animal case has been reported since 2005 and 2014, respectively. Military personnel is frequently exposed to outdoor environment, we hypothesized that their risk of exposure to wild animal is higher than general population. This study aimed to determine the level of rabies risk exposure and the adequacy of treatment by analyzing animal-bitten patients and assessing the status of post-exposure prophylaxis(PEP).

**Methods:**

This study was conducted from January 1, 2012, to December 31, 2019, in 18 military hospitals across the country. The patients with animal bite-related diagnoses were included in the study. The diagnoses were made based on the ICD-10 code related to animal bite. We excluded patients with human bites, insect bites, and snake bites. Cases of repeated visits for additional vaccination were excluded as duplicate cases.

**Results:**

A total of 330 cases of animal bites were included. No human rabies was reported. 297 (90%) were military personnel, with a males (92.1%) and a mean age of 32±11 years. The most frequent site of bite was the hands and fingers (69.7%), followed by the legs (12.4%) and arms (3.9%). Dogs were responsible for 44.8% of bites, followed by cats (38.5%) and mice (5.8%). Empirical antibiotic was administered to 58.5%, while tetanus vaccination was given to 22.1%. In terms of PEP, the matching rate with domestic health authorities’ standards was 62.1%. Category II was the most common criteria for bite injuries (71.5%), according to the World Health Organization(WHO)’s criteria.

**Figure:**
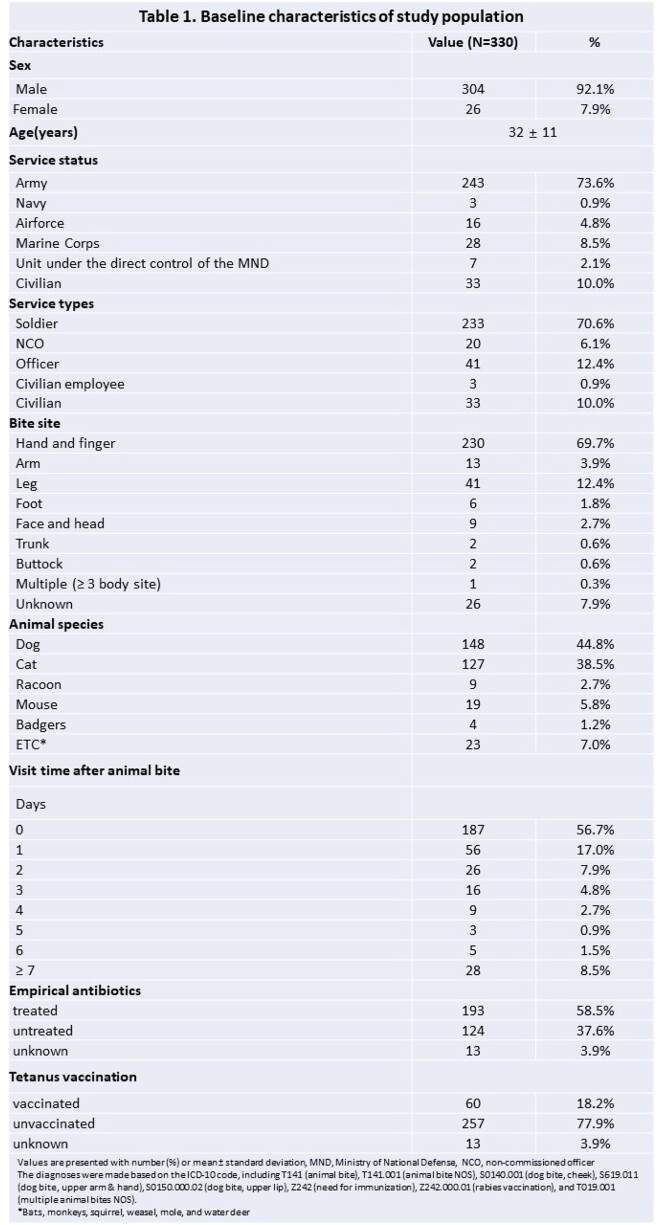

**Figure:**
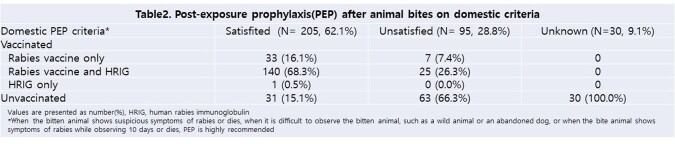

**Conclusion:**

Dogs and cats are the most frequently bitten animals, as in previous studies, and hands and fingers, were the most frequently bitten. We postulate that bites occur mainly during contact with animals using hands, suggesting that precaution is necessary. PEP requires immediate medical attention, however 43.7% of patients visited hospitals more than 24 hours after the bite, indicating a need to improve awareness of the disease. PEP was not performed in 15.1% of cases despite meeting domestic standards, and 37.3% in the WHO’s criteria. These emphasize that efforts should be needed to provide appropriate PEP that meets the relevant criteria and minimize unnecessary use of medical resources.

**Disclosures:**

**All Authors**: No reported disclosures

